# Microstructure and Properties of Mechanical Alloying Al-Zr Coating by High Current Pulsed Electron Beam Irradiation

**DOI:** 10.3390/nano10122398

**Published:** 2020-11-30

**Authors:** Xiangcheng Li, Huiru Liu, Nana Tian, Conglin Zhang, Peng Lyu, Qingfeng Guan

**Affiliations:** 1 School of Materials Science and Engineering, Jiangsu University, Zhenjiang 212013, China; 2221905031@stmail.ujs.edu.cn (X.L.); 3180702060@stmail.ujs.edu.cn (H.L.); 2112005004@stmail.ujs.edu.cn (N.T.); lvp@ujs.edu.cn (P.L.); 2 School of Material Science and Engineering, Yancheng Institute of Technology, Yancheng 224051, China; zhangcl@ycit.cn

**Keywords:** high-current pulsed electron beam (HCPEB), zirconium alloying, microstructure, surface hardness, corrosion resistance

## Abstract

The “HOPE-I” type high-current pulsed electron beam (HCPEB) equipment was used to irradiate the pure aluminum material with Zr coating preset by ball milling to realize the alloying of a Zr–Al coating surface. The microstructure and phase analysis were conducted by XRD, SEM, and TEM. The experimental results show that after Zr alloying on the Al surface by HCPEB, a layer of 15 μm was formed on the surface of the sample, which was mainly composed of Zr and Al–Zr intermetallic compounds. A large number of Al_3_Zr (Ll2) particles was uniformly distributed in the alloyed layer, and the Al grains were obviously refined. In addition, the surface hardness and corrosion resistance of the samples were improved significantly after HCPEB irradiation.

## 1. Introduction

Aluminum (Al) and its alloys have been widely used in industrial production because of their excellent mechanical and physical properties, such as being lightweight; having high specific strength, good ductility, and low density; and having excellent electrical and thermal conductivity. However, poor surface properties such as low hardness, poor wear resistance, and corrosion resistance impede their potential wide-spread use. Depositing a protective layer with excellent adhesion, high strength, and corrosion resistance on the surface of the aluminum alloy is an effective way to improve the performance of the material. The commonly used methods of surface modification for aluminum alloys are electroplating [1], chemical plating [2], anodic oxidation [3], vapor deposition [4], thermal spraying [5], etc., which have achieved remarkable results in their respective practical applications. However, there are some problems, such as thin and brittle coatings, oxide film discontinuities, and large surface roughness.

At the same time, many researchers have been focusing on surface alloying, which they hope will improve the overall performance of the materials [6,7]. Zr is an indispensable element in many high-performance aluminum alloys that can not only increase the recrystallization temperature and strength, but also refine the structure of aluminum alloys. In addition, Zr can improve the hardness and the stress corrosion resistance of the alloy [8]. There is only one kind of Al_3_Zr (Ll2) phase in the intermetallic compound when the Zr content is less than 53at%. The Ll2 phase not only plays the role of dispersion strengthening and sub-crystalline strengthening, but also reduces the plastic deformation of the aluminum alloy and effectively inhibits the recrystallization during subsequent heat treatment [9]. It is an “effective” phase that improves the overall performance of aluminum alloys [10,11]. However, in the solidification process of Al–Zr, it is difficult to reach equilibrium in the melting and casting process. Zr elements are easily segregated to form coarse primary Al_3_Zr phases, and the precipitate dilution zone is easily formed near grain boundaries and interdendritic regions [12,13], which has caused great harm to the alloy. It was demonstrated that these problems can be ameliorated by using non-equilibrium state processing techniques such as mechanical alloying (MA), but MA does not significantly improve the corrosion resistance, friction, and wear properties of the material [14,15,16,17].

As a new surface alloying technology with great potential, high-current pulsed electron beam (HCPEB) has been applied in surface treatment for years [18,19,20]. Under HCPEB irradiation, the high beam energy (10^8^–10^9^ W/cm^2^) is transferred into the surface layer instantaneously, which leads to extremely rapid heating and melting (even evaporating), followed by rapid solidification (10^7^–10^9^ k/s). The surface composition of the material is homogenized and nanocrystals are formed due to its ultra-high temperature gradient (10^8^ K/s). In addition, the sub-surface layer of the material rapidly undergoes plastic deformation and forms a rich structure of points, lines, surfaces, and other defects because of the thermal stress generated by thermal shock [21]. The alloying that occurs between the mutually insoluble elements improves the comprehensive performance of the material surface after HCPEB irradiation, which cannot be achieved by conventional surface treatment techniques. 

In this paper, the high-current pulsed electron beam was used to conduct Zr alloying treatment on a pure aluminum surface to improve its surface properties, such as strength and corrosion resistance. The microstructure of the alloying layer was characterized to explore the mechanism of Al–Zr alloying and its performance improvement mechanism after HCPEB.

## 2. Materials and Methods 

### 2.1. Specimen Preparations and Zr Coating on Al

The as-received pure Al was machined into square plates with dimensions of 10 × 10 × 5 mm. All the samples were ground (using SiC abrasive paper), polished (with 1 mm diamond paste), and cleaned ultrasonically in acetone. The Zr powder (99.99% in purity with about 75~80 μm particle size) was blended for 1 h in a ball mill with a weight ratio of 10 Al to 1 Zr. For full mixing, the milling speed was 250 r min^−1^ and a high-purity argon was used as the shielding gas to prevent oxidation and pollution.

The “HOPE-I”-type HCPEB equipment was adopted to conduct the surface alloying treatment on the pre-treated Al, and the number of irradiations was 10, 20, 30, and 40. The process parameters of HCPEB irradiation were as follows: the electron energy was 27 keV, the current pulse duration was 1.5 μs, the energy density was 4 J/cm^2^, the beam diameter was 60 mm, the pulse interval was 10 s, and the vacuum as 5.0 × 10^−3^ Pa.

### 2.2. Microstructural Characterization

Phase identification was conducted on a Rigaku D/max-2500/PC X-ray (XRD) diffraction, and the microstructural evolution and phase composition of the overlay was comprehensively studied using a JEOL JSM-7100F scanning electron microscope (SEM) with an Inca energy 350 energy dispersive spectrometer (EDS). In order to further clarify the resultant phase and the induced microstructural modification within the alloying layer, transmission electron microscope (TEM) observation on a JEM-2100 high-resolution microscope at 15 kV accelerating voltage was used. The preparation process of the alloying layer for TEM observation was as follows: single-side milling, pits, and ion thinning.

### 2.3. Properties Testing

Microhardness was measured on an HVS-1000 microhardness measurement device with a load of 0.147 N (15 g) for 8 s. Seven test points were installed in each sample in order to reduce random error. The test result was the average value of the middle five readings (the maximum and minimum values were removed). The polarization curve and impedance spectrum of each sample was measured using the Bio-Logic VMP2 electrochemical workstation. The whole setup was connected to a conventional three-electrode cell, including a working electrode, a saturated calomel electrode (SCE) as the reference electrode, and a platinum sheet as the counter electrode. The electrolyte solution was 3.5 wt% NaCl (0.6 M). The cyclic polarization (CP) was done at a sweep rate of 0.333 mV/s. To avoid experimental errors, the other 5 surfaces (except the tested surface) were all sealed with vulcanized silicone rubber.

## 3. Results and Discussion

### 3.1. Microstructure Characterization

**Figure 1:**
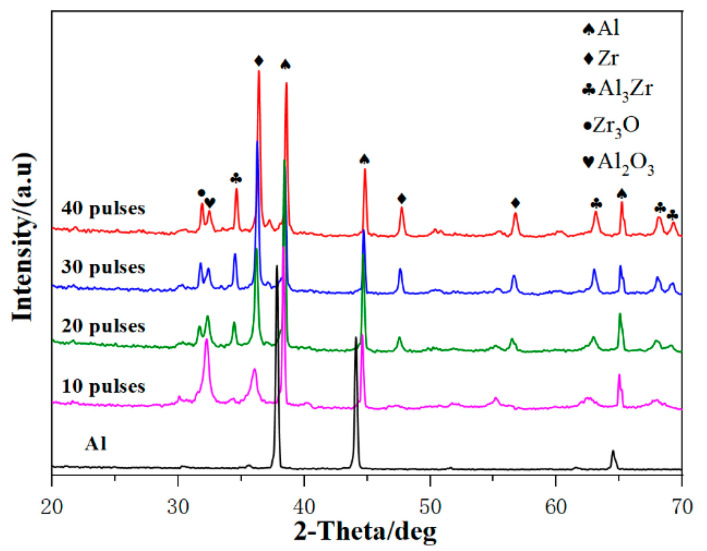
The XRD analysis of the samples before and after alloying.

Figure 1 shows the XRD patterns of the samples after HCPEB irradiation with different pulses. Three diffraction peaks of Al, Zr, and Al_3_Zr can be seen on the sample surface after 10-pulsed irradiation, which indicates that Al reacted with Zr and produced the Al_3_Zr phase. As the number of irradiation pulses increased to 20, the intensity of the diffraction peak of Zr decreased significantly while that of Al_3_Zr increased slightly. This indicates that Zr particles were gradually melted into the matrix and more Al_3_Zr intermetallic compounds were formed between Al–Zr. After 30-pulsed irradiation, the diffraction peak intensity of Al_3_Zr was further increased. However, when the pulse number continued to increase up to 40, the diffraction peak intensity of Al_3_Zr was no more significant than 30, which indicates that Al_3_Zr was not further formed. Therefore, it can be concluded that the content of Al_3_Zr in the irradiated surface layer increased when the pulse number was increased to 30. 

**Figure 2:**
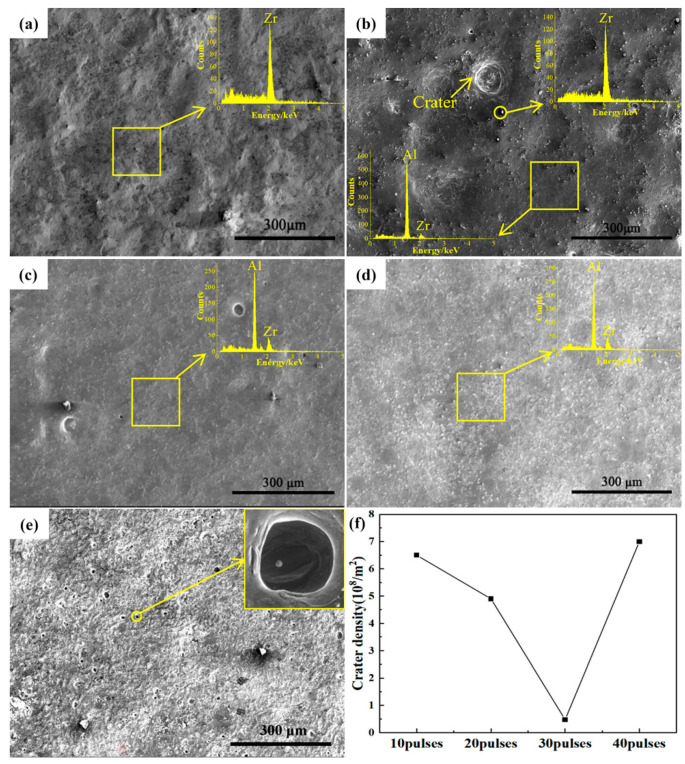
Surface SEM images of samples before and after HCPEB irradiation: (**a**) milled samples, (**b**) 10 pulses, (**c**) 20 pulses, (**d**) 30 pulses, (**e**) 40 pulses, and (**f**) crater density of different pulses.

Figure 2a demonstrates the surface morphology and EDS spectra of the original sample after ball milling preparation. The image shows that the surface layer of the original sample after ball milling is rough and the main component of the sample surface can be concluded to be Zr. 

Figure 2b–e consists of SEM images of the sample surface treated with 10-, 20-, 30-, and 40-pulsed irradiation. From Figure 2b, for the 10-pulsed sample there were a few craters on the surface of the sample, which were formed due to the partial eruption from the melted subsurface layer caused by irradiation. Abundant particles with a size of about 5 μm were observed on the surface of the sample, which is evidenced as pure Zr particles from the data results of the EDS point scanning. The results of the EDS data on the homogeneous microstructure of the sample surface show that the Zr content is 14.7 at%, which means that part of the Zr element had been dissolved into the matrix. Figure 2c shows the surface morphology of the sample after 20-pulsed irradiation. It can be seen that the large craters on the surface basically disappeared, and the remaining small craters were the result of gradually repaired large craters as the number of irradiation pulses increased [22,23,24]. From the EDS result, the Zr content of the sample surface in this area reached 33.7 at%, which illustrates that more Zr dissolved into the surface layer with the increment of the pulse number. Figure 2d is the surface morphology of the sample after 30-pulsed irradiation, which shows that the craters on the surface of the sample have almost disappeared. Additionally, the EDS result shows that the Zr content on the surface of the sample had increased to 35.1 at%, which showed no significant change compared with that of the 20-pulsed sample. After 40-pulsed irradiation, as shown in Figure 2e, the condition of the surface of the sample deteriorated significantly and the number of small craters increased dramatically, which was due to a large number of former Al_3_Zr particles becoming the core for the formation of new melt craters. 

The density of the craters of different pulses was charted as Figure 2f. It can be concluded that the crater density on the surface of the sample after electron beam irradiation decreases initially, reaching a minimum at 30-pulsed irradiation and then increasing again. In addition, when the pulse number reached 30, Zr-rich particles decreased and the Zr content increased where the microstructure was homogeneous. Therefore, this indicates that when the irradiation pulse number is smaller than 30, increasing the pulse number is beneficial to the homogeneous distribution of Zr elements on the substrate surface.

**Figure 3:**
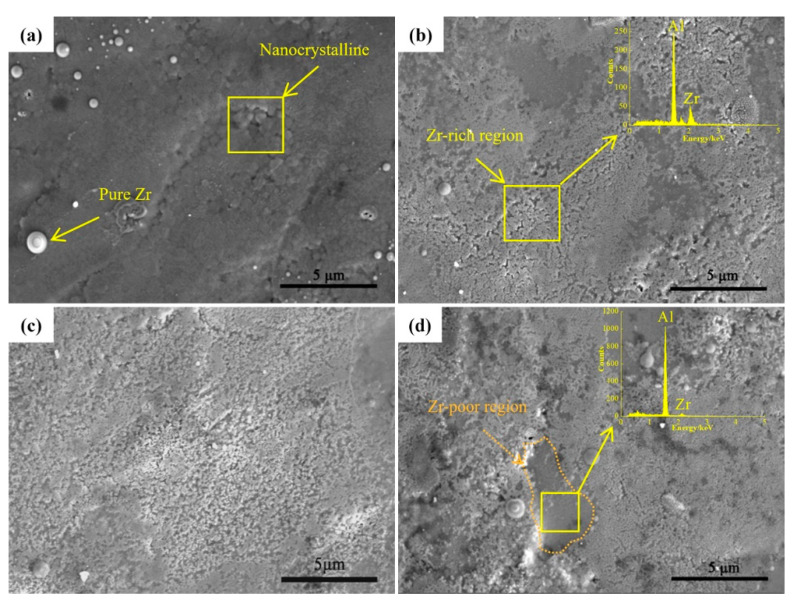
Surface SEM images of samples after HCPEB irradiated: (**a**) 10 pulses, (**b**) 20 pulses, (**c**) 30 pulses, and (**d**) 40 pulses.

Figure 3 is a high-magnification SEM image of the sample surface under different pulses. From Figure 3a, nanoscale grains, a common structure of metallic materials after HCPEB treatment, appeared on the surface of the Al–Zr alloyed layer after 10-pulsed irradiation. The formation of this structure is related to the extremely fast heating and the subsequent instant solidification during the process of HCPEB treatment [25]. Figure 3b shows the surface morphology of the sample after 20-pulsed irradiation. The EDS result shows that the surface of the sample contains Al and Zr elements, which means that when the number of irradiations was increased to 20, the Zr particles dissolved into the matrix material and the dense Zr-rich region appeared on the sample surface. 

When the number of pulses was further increased to 30, as shown in Figure 3c, the surface of the sample was homogeneous and Zr-poor areas were absent. However, it can be seen in Figure 3d that when the number of pulses increased to 40, the distribution of the Zr-rich regions tended to be non-uniform and Zr-poor regions appeared in some areas. This is probably because the excessive number of pulses caused excessive stress on the surface of the specimen, resulting in deterioration of the surface quality and exfoliation of the Zr coating in some areas.

**Figure 4:**
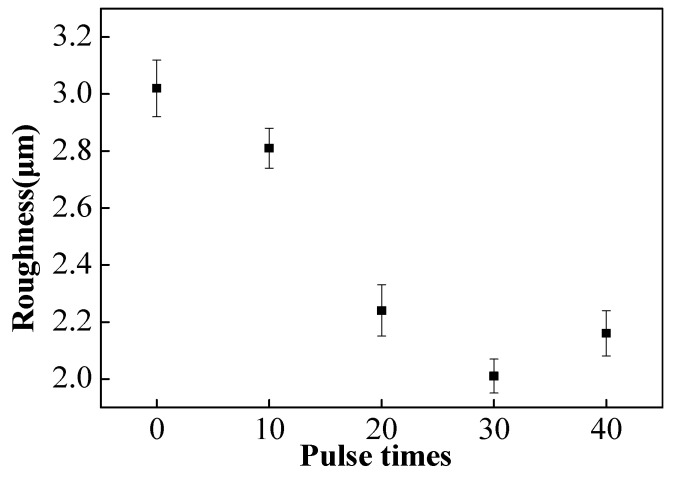
The surface roughness of samples before and after HCPEB irradiation.

Figure 4 shows the roughness (Ra) value analysis of the sample surface before and after HCPEB irradiation treatment. It can be seen that with the increase in the number of irradiations, there was a significant change in the roughness of the samples, with 3.02 μm for the initial sample and 2.81 μm, 2.24 μm, 2.01 μm, and 2.16 μm for samples irradiated for 10, 20, 30, and 40 pulses, respectively. It is worth noting that the 30-pulsed samples have the lowest surface roughness value. Besides, when the number of HCPEB irradiations was less than 30, the roughness value of the sample surface decreased significantly as the number of irradiations increased. This was due to the gradual dissolution of the Zr particles in the matrix and the decrease or even disappearance of the number of craters on the sample surface. However, when the number of irradiations was increased to 40, the roughness value of the sample surface grew, which was related to the deterioration of the surface condition of the sample surface, the appearance of a large number of small craters, and the formation of a Zr-poor region, as described above. As a result, an alloy layer with a good surface condition can be obtained after 30-pulsed irradiation.

**Figure 5:**
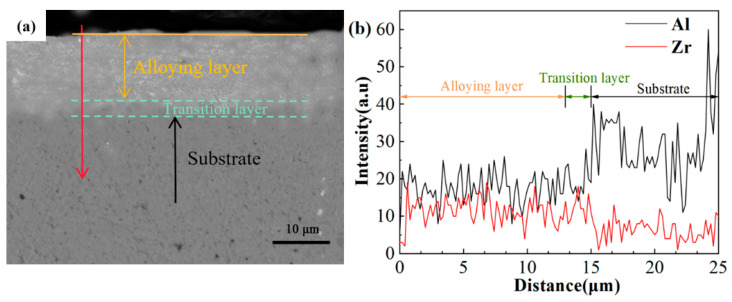
Cross-section EDS images of the 30-pulsed sample: (**a**) the BSE image of the cross-section and (**b**) the result of EDS line scan.

Considering the better surface condition, the samples with 30 pulses were chosen to investigate the following experiment and the cross-sectional morphology shown in Figure 5. 

Figure 5a shows an electron back scattered diffraction (BSE) image of the cross-section of the sample after 30-pulsed irradiation. The light gray layer, distinctly different from the substrate structure, was formed on the surface with an average thickness of about 15 μm, including an alloy layer and a transition layer. Figure 5b shows the energy dispersive spectroscopy (EDS) line scan along the direction of the red arrow in Figure 5a. It shows that the depth of the Zr-enriched layer was up to about 15 μm. It indicated that the Zr elements were well mixed and fused with the pure aluminum matrix. A very thin (1–2 μm) transition layer can be seen formed between the alloying layer. The substrate can also be seen formed, and this, combined with the alloying layer, composed the melted layer. 

Combined with cross-sectional topography and EDS line scan analysis, a relatively thick Zr-rich alloy layer was produced on the pure Al surface after 30-pulsed irradiation.

**Figure 6:**
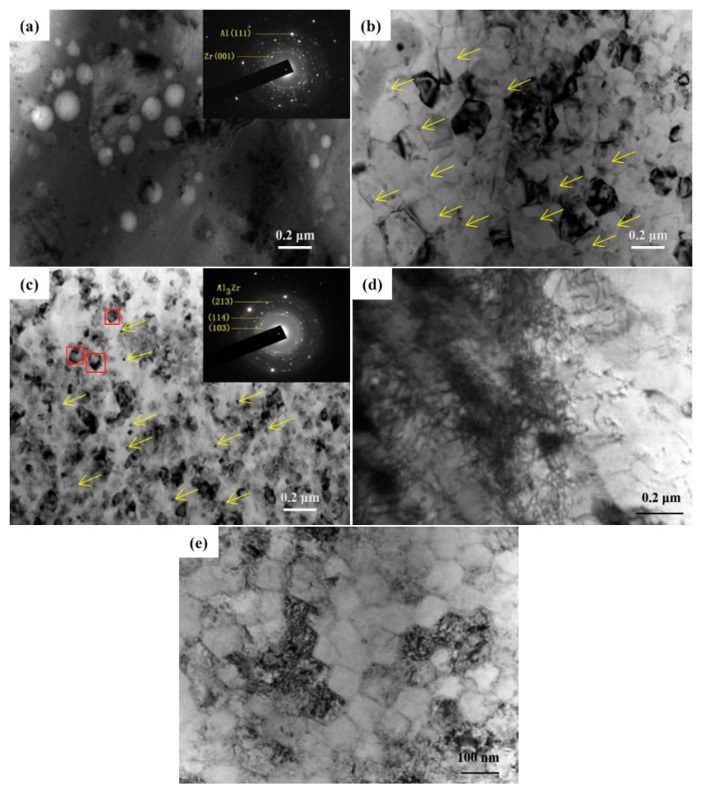
TEM micrographs of the alloying layer after HCPEB irradiation: (**a**) 10 pulses, (**b**) 20 pulses, (**c**) 30 pulses, and (**d**–**e**) high-density dislocation lines and ultra-fine grain after 30-pulsed irradiation.

Figure 6 shows the TEM images of the alloying layers of Al–Zr samples after 10-, 20-, and 30-pulsed irradiation. In the 10-pulsed sample, white particles about 0.2 μm in size were observed on the surface of the sample. By analyzing the pattern of the selected area electron diffraction (SAED) in Figure 6a, these tiny white particles were identified as Zr particles. 

Figure 6b is the TEM image of the sample after 20-pulsed irradiation. The Zr particles of large size disappeared and the Al grains were significantly refined to sizes between 0.1~0.2 μm. A small amount and fine-sized (≤20 nm) particles were distributed in the Al matrix, which was due to the presence of Zr in the Al matrix as a solid solution, resulting in a small number of precipitated phase particles. 

After 30-pulsed irradiation, as shown in Figure 6c, a large amount of diffusely distributed grey nanoparticles was generated on the surface of the material, which had an average size of about 40 nm. The SAED analysis showed that these particles were Ll2-type Al_3_Zr phases. This phenomenon was caused by the high energy injected into the system with electron beam irradiation, which promoted the dispersed precipitation of Zr elements from the Al–Zr supersaturated solid solution, resulting in the formation of the nanoscale sub-stable Al_3_Zr phases (about 30–40 nm).

Kyoung [26] showed that the Ll2-type Al_3_Zr phase is the best form of Zr existing in aluminum alloys, and its diffuse and uniform distribution in the matrix contributes to the improvement of mechanical properties and corrosion resistance of the alloy. When irradiated, the surface layer of the alloy was melted rapidly due to the high-speed deposition of the energy. During the rapid solidification, there was a large amount of Al_3_Zr nucleating at the Al grain boundaries, thus preventing the Al grains from growing and eventually obtaining fine Al grains.

Figure 6d–e shows TEM images of the sample surface after 30-pulsed irradiation, which demonstrates some crystal defects and ultrafine crystal structures in the alloy layer. 

As shown in Figure 6d, the high-density of dislocation structures were formed in the sample alloying layer. HCPEB irradiation induced large stress in the sample surface layer, leading to the formation of a high-density dislocation structure around the Al_3_Zr particles that hindered the growth of Al grains and ultimately promoted the refinement of the surface Al grains [27]. Figure 6e shows the ultrafine-grained structures of the sample surface layer after electron beam irradiation. These ultrafine-grained structures provided the channel for the diffusion of Zr atoms in the Al matrix and promoted the solid solution of Zr in Al, which is conducive to the preparation of high-quality alloyed layers on the sample surface [28,29].

### 3.2. Microstructure Characterization

**Figure 7:**
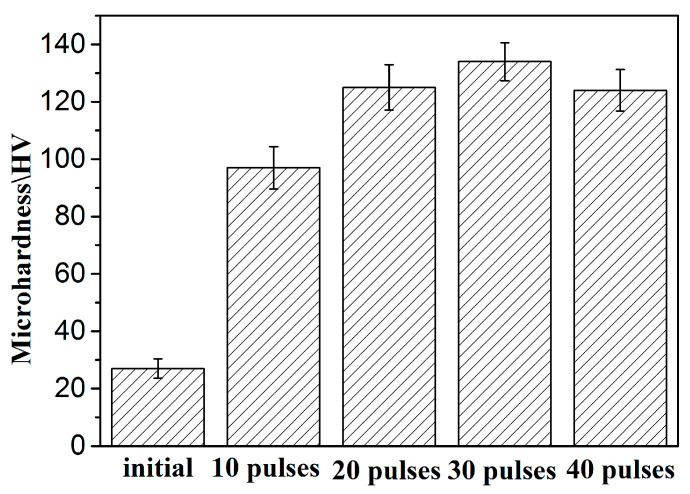
Microhardness measurements of the alloyed sample before and after HCPEB irradiation.

Figure 7 shows the microhardness values of all samples (including before and after HCPEB irradiated and alloyed samples). The value of the original sample was about 27 HV. After 30-pulsed irradiation, the value of the samples reached about 134 HV, which was the maximum value of HCPEB alloyed samples. However, the microhardness decreased after 40-pulsed irradiation. 

According to various studies, the improvement of hardness was attributed to several factors, as follows:

- The main strengthening mechanism of samples irradiated with 10 pulses was the dispersion strengthening of nanoscale Zr particles.
- After 20 and 30 pulses of irradiation, the Al grains were significantly refined, and the solid solubility of Zr in Al was greatly improved. Therefore, the fine-grain strengthening and solid-solution strengthening were the main strengthening mechanisms at this time.
- The HCPEB alloyed sample showed well dispersed Al_3_Zr phases. Moreover, the defect structures and nano gains were also observed in the alloying layer after 30-pulsed irradiation. Thus, the combined effects of the nanoscale Al_3_Zr phases, nanograins, and defect structures are responsible for the hardness improvement for 30-pulsed irradiated samples.
- The decrease of the hardness after 40-pulsed irradiation was related to the exfoliation of the Zr coating and the release of residual stresses [30].

### 3.3. Corrosion Performance Analysis

**Figure 8:**
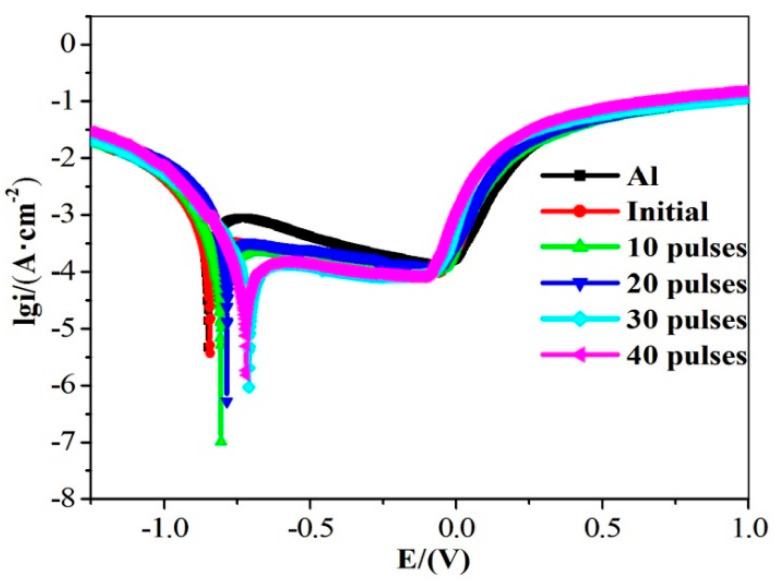
Corrosion polarization curves of pure Al and samples before and after HCPEB irradiation.

The potentiodynamic polarization curves of the samples before and after HCPEB irradiation in 3.5 wt% NaCl solution are shown in Figure 8. Parameters such as corrosion current (I_corr_) density and corrosion potential (E_corr_) were calculated and tabulated in Table 1. It can be seen in Figure 8 and Table 1 that the self-corrosion potential (E_corr_) increased, whereas the self-corrosion current (I_corr_) decreased after the HCPEB irradiation. This means that Zr alloying can effectively slow down the dissolution rate of the anodic activity of the samples and improve the corrosion resistance of the Al–Zr alloy layer in 3.5 wt% NaCl solution. The I_corr_ value of the 30-pulsed irradiation was 0.443 μA/cm^2^, which is lower than the pure Al and ball-milled original sample and has the best corrosion resistance. However, the I_corr_ of the 40-pulse irradiated samples was slightly higher than that of the 30-pulse irradiated samples, which was related to the deterioration of the surface condition and the increase in the crater numbers after 40-pulsed irradiation. 

Overall, corrosion resistance is significantly improved after HCPEB irradiation. The main reasons are summarized as follows:

- After HCPEB irradiation, high-density crystal defects were formed inside the crystal grains. A large number of crystalline defects can provide a channel for oxygen ions to enter the sample, resulting in the formation of a dense Al_2_O_3_ film on the surface, which improves the corrosion resistance of the sample [31].
- After HCPEB irradiation, the Al grains were significantly refined. Wang et al. [27] found that for Al and its alloys, when the grains are refined, the number of active atoms on the sample surface increases, which can promote the generation of a passivation film on the sample surface and thus improve the corrosion resistance of the material. Therefore, HCPEB alloying treatment is very helpful in improving the corrosion resistance of the sample surface.
- After 30-pulsed irradiation, a large number of diffuse and uniformly distributed Ll2-type Al_3_Zr phases was formed on the sample surface. This helped to improve the corrosion resistance of the sample surface [11].

**Figure 9:**
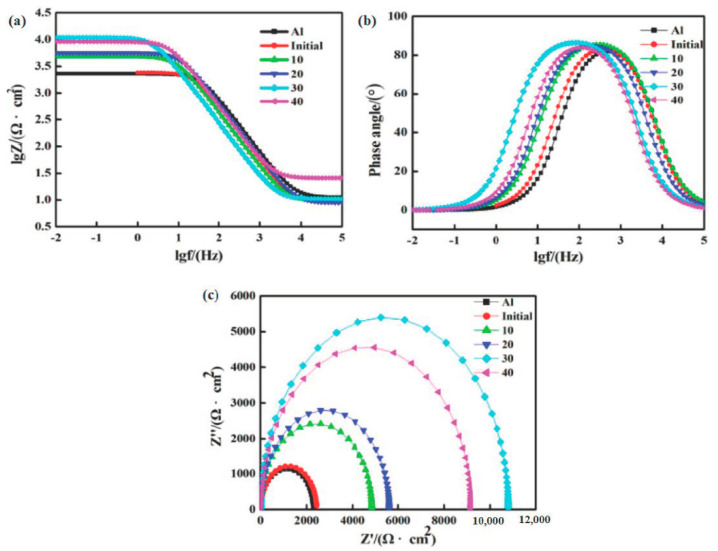
EIS of samples in 3.5 wt% NaCl before and after HCPEB alloying of Zr: (**a**) impedance magnitude and frequency, (**b**) phase angle and frequency, and (**c**) Nyquist plot.

The electrochemical impedance spectra (EIS) measurements performed in 3.5 wt% NaCl solution of pre- and post-treatment by HCPEB irradiation are shown in Figure 9, which displays the relationship among impedance magnitude |Z|, phase angle, and frequency. From Figure 9a, the impedance magnitude of the sample after HCPEB irradiation in the low-frequency region is higher than that of both the pure Al and the ball-milled original sample, which indicates that the corrosion resistance of the samples was improved. The samples with 30-pulsed irradiation had the highest impedance magnitude and slope in the mid-frequency region of the curve. The relationship of the phase angles and lg f is shown in Figure 9b. The phase angles of alloyed samples are evidently bigger than the unalloyed sample in the low frequency scopes, especially for the 30-pulsed sample. From the Nyquist plot in Figure 9c, it can be seen that the capacitive arcs of the irradiated samples are larger than those before irradiation, which means that the HCPEB alloying treatment improved the corrosion resistance of the samples.

In summary, the results of the EIS analysis further support the benefits of HCPEB alloying for improving the corrosion resistance of materials.

## 4. Conclusions

This paper presents a detailed analysis of the microstructure evolution and performance changes occurring in the alloying layer of Zr–Al coatings prepared by ball milling under different parameters of HCPEB irradiation. The main findings are summarized as follows:

- The HCPEB surface alloying treatment promoted the solid solution of Zr elements in the Al matrix and achieved Zr alloying on the surface of the Al matrix, forming a Zr–Al alloying layer of several micrometers. Among them, 30-pulsed irradiation can produce an alloying layer with a relatively good surface condition on the sample surface.
- After the HCPEB irradiation, the surface of the samples showed obvious grain refinement and uniform composition distribution. In addition, a large number of Ll2-type Al_3_Zr phases with small size and diffuse uniform distribution appeared in the samples after 30-pulsed irradiation.
- Surface microhardness of samples treated by HCPEB irradiation improved significantly, which was mainly the result of fine crystal strengthening, solid solution strengthening, dispersion strengthening, and dislocation strengthening. Among them, 30-pulsed irradiation of the sample surface hardness was the highest.
- After HCPEB surface alloying, the corrosion resistance improved dramatically due to the crystal defects, significant refinement of Al grains, and generation of a diffusely distributed Al_3_Zr phase on the top of the surface. The corrosion resistance of the samples after 30-pulsed irradiation treatment was the best.

## Figures and Tables

**Table 1:** The corrosion data of the alloyed Al–Zr and unalloyed pure Al samples.

Sample	E_corr_/V	I_corr_/(μA·cm^−2^)
Al	−0.873	2.786
original sample	−0.860	2.710
Al–Zr 10 pulses	−0.623	0.821
Al–Zr 20 pulses	−0.605	0.622
Al–Zr 30 pulses	−0.591	0.443
Al–Zr 40 pulses	−0.584	0.450

## References

[1] Karunakaran M., Pugazh Vadivu M. (2019). Magnetic and micro-mechanical behavior of Cu-Ni-P-W-TiO_2_ hybrid composite electroplating on Al alloy substrate. J. Magn. Magn. Mater..

[2] Kang R., Peng Z., Liu B., Wang D., Liang J. (2017). A protocol for fast electroless Ni-P on Al alloy at medium-low temperature accelerated by hierarchically structured Cu immersion layer. Surf. Coatings Technol..

[3] Wang L.S., Bu Z.X., Lu M., Geng Y., Chen M.H., Sun L. (2017). Thick oxide coatings formed by spark anodizing of Mg-Al alloy in alkaline phosphate-silicate electrolytes. J. Alloys Compd..

[4] Sun W., Wang L., Yang Z., Li S., Wu T., Liu G. (2017). Fabrication of polydimethylsiloxane-derived superhydrophobic surface on aluminium via chemical vapour deposition technique for corrosion protection. Corros. Sci..

[5] Wang Q., Sun Q., Zhang M.-X., Niu W.-J., Tang C.-B., Wang K.-S., Rui X., Zhai L., Wang L. (2018). The influence of cold and detonation thermal spraying processes on the microstructure and properties of Al-based composite coatings on Mg alloy. Surf. Coat. Technol..

[6] Phanikumar G., Dutta P., Galun R., Chattopadhyay K. (2004). Microstructural evolution during remelting of laser surface alloyed hyper-monotectic Al–Bi alloy. Mater. Sci. Eng. A.

[7] Chen S., Zhao J. (2013). Solidification of monotectic alloy under laser surface treatment conditions. Acta Metall. Sin..

[8] Li H., Liang X., Li F., Guo F., Li Z., Zhang X. (2007). Effect of Y content on microstructure and mechanical properties of 2519 aluminum alloy. Trans. Nonferr. Met. Soc. China.

[9] Nayak S.S., Murty B.S. (2003). Synthesis of nanocrystalline L12-Al3Zr and Al3Ti by mechanical alloying. Trans. Indian Inst. Met..

[10] Fang L., Zhang Z., Fang H., Huang L., Chen K. (2019). Effects of Si additions on the precipitation evolution of dilute Al-Zr-Yb alloys. Mater. Charact..

[11] Tsivoulas D., Robson J.D. (2015). Heterogeneous Zr solute segregation and Al3Zr dispersoid distributions in Al–Cu–Li alloys. Acta Mater..

[12] Hu H., Zhao M., Wu X., Jia Z., Wang R., Li W., Liu Q. (2016). The structural stability, mechanical properties and stacking fault energy of Al3Zr precipitates in Al-Cu-Zr alloys: HRTEM observations and first-principles calculations. J. Alloys Compd..

[13] Jiao L., Zhao Y.-T., Chen J.-C., Chen L. (2016). Microstructure and properties of Al3Zr/2024Al in situ composites after forging. Rare Met..

[14] Luzin V., Spencer K., Zhang M.X. (2011). Residual stress and thermo-mechanical properties of cold spray metal coatings. Acta Mater..

[15] Hao L., Lu Y., Sato H. (2013). Influence of Metal Properties on the Formation and Evolution of Metal Coatings during Mechanical Coating. Metall. Mater. Trans. A.

[16] Fogagnolo J., Velasco F., Robert M., Torralba J. (2003). Effect of mechanical alloying on the morphology, microstructure and properties of aluminium matrix composite powders. Mater. Sci. Eng. A.

[17] Suryanarayana C., Ivanov E., Boldyrev V. (2001). The science and technology of mechanical alloying. Mater. Sci. Eng. A.

[18] Lyu P., Chen Y., Liu Z., Peng C.T., Cai J., Zhang C., Jin Y., Guan Q. (2019). The effect of high current pulsed electron beam irradiation on microstructure and properties Cu-Fe powder metallurgical alloys. Mater. Res. Express.

[19] Zhang C., Lv P., Xia H., Yang Z., Konovalov S., Chen X., Guan Q. (2019). The microstructure and properties of nanostructured Cr-Al alloying layer fabricated by high-current pulsed electron beam. Vacuum.

[20] Zhang L., Peng C.T., Guan J., Lv P., Guan Q., Lu R. (2019). Nanocrystalline Cr-Ni alloying layer induced by high-current pulsed electron beam. Nanomaterials.

[21] Dong S., Zhang C., Zhang L., Cai J., Lv P., Jin Y., Guan Q. (2018). Microstructure and properties of Cu-Cr powder metallurgical alloy induced by high-current pulsed electron beam. J. Alloys Compd..

[22] Zhang C., Cai J., Lv P., Zhang Y., Xia H., Guan Q. (2017). Surface microstructure and properties of Cu-C powder metallurgical alloy induced by high-current pulsed electron beam. J. Alloys Compd..

[23] Xia H., Zhang C., Lv P., Cai J., Jin Y., Guan Q. (2018). Surface alloying of aluminum with molybdenum by high-current pulsed electron beam. Nucl. Instrum. Methods Phys. Res. Sect. B Beam Interact. Mater. Atoms.

[24] Chernov I.P., Ivanova S.V., Krening M.K., Koval N., Larionov V.V., Lider A.M., Pushilina N.S., Stepanova E.N., Stepanova O.M., Cherdantsev Y.P. (2012). Properties and structural state of the surface layer in a zirconium alloy modified by a pulsed electron beam and saturated by hydrogen. Tech. Phys..

[25] Zhang C., Lv P., Cai J., Zhang Y., Xia H., Guan Q. (2017). Enhanced corrosion property of W-Al coatings fabricated on aluminum using surface alloying under high-current pulsed electron beam. J. Alloys Compd..

[26] Moon K.I., Chang K.Y., Lee K.S. (2000). The effect of ternary addition on the formation and the thermal stability of L12 Al3Zr alloy with nanocrystalline structure by mechanical alloying. J. Alloys Compd..

[27] Wang R.-N., Tang B.-Y., Peng L.-M., Ding W.-J. (2012). Ab initio study of the effect of Zr content on elastic and electronic properties of L12–Al3(Sc1−xZrx) alloys. Comput. Mater. Sci..

[28] Li G.R., Zhao Y.T., Wang H.M., Chen G., Dai Q.X., Cheng X.N. (2009). Fabrication and properties of in situ (Al _3_Zr + Al_2_O_3_) p/A356 composites cast by permanent mould and squeeze casting. J. Alloys Compd..

[29] Kumar Makineni S., Sugathan S., Meher S., Banerjee R., Bhattacharya S., Kumar S., Chattopadhyay K. (2017). Enhancing elevated temperature strength of copper containing aluminium alloys by forming L12 Al3Zr precipitates and nucleating θ″ precipitates on them. Sci. Rep..

[30] Tian N., Li S., Zhang C., Cai J., Lyu P., Konovalov S., Chen X., Peng C.T., Guan Q. (2019). The surface modification of aluminum by mechanical milling of Pb coating and high current pulsed electron beam irradiation. Mater. Res. Express.

[31] Diankun L., Bo G., Guanglin Z., Jike L., Liang H. (2017). High-Current Pulsed Electron Treatment of Hypoeutectic Al–10Si Alloy. High Temp. Mater. Process..

